# The Operational Performance of an Ultrafiltration Pilot Unit for the Treatment of Ultra-Concentrated Brines

**DOI:** 10.3390/membranes14120276

**Published:** 2024-12-20

**Authors:** Giuseppe Scelfo, Paula Serrano-Tari, Ritamaria Raffaelli, Fabrizio Vicari, Isabel Oller, Andrea Cipollina, Alessandro Tamburini, Giorgio Micale

**Affiliations:** 1 Department of Engineering, Università degli Studi di Palermo, 90128 Palermo, Italy; giuseppe.scelfo02@unipa.it (G.S.); alessandro.tamburini@unipa.it (A.T.); giorgiod.maria.micale@unipa.it (G.M.); 2 ResourSEAs SrL, 90141 Palermo, Italy; fabrizio.vicari@resourseas.com; 3 CIESOL (Centro de Investigaciones de la Energía Solar), Join Centre of the University of Almería-CIEMAT, 04120 Almería, Spain; pserrano@psa.eu (P.S.-T.); iollier@psa.eu (I.O.); 4 Plataforma Solar de Almería-CIEMAT, 04200 Tabernas, Spain

**Keywords:** bittern, ultrafiltration, viscosity, fouling, membranes

## Abstract

The valorization of ultra-concentrated seawater brines, named bitterns, requires preliminary purification processes, such as membrane filtration, before they can be fully exploited. This study investigates the performance of an ultrafiltration pilot plant aimed at separating organic matter and large particles from real bitterns. An empirical model for the bittern viscosity was developed to better characterize the membrane. Distinct variations in permeability, fouling resistance and rejection coefficient were observed under operational pressures ranging from 2 to 4 bar. Working at low pressure (2 bar), the pilot plant achieves permeability and rejection coefficient values of 17 L/m^2^hbar and 95%, respectively. Foulant behavior was characterized by determining a “fouling resistance”, obtaining an average value of $10^{13}$ m^−1^. Tests with three distinct bittern samples were conducted to assess the influence of chemical composition and organic matter content on membrane permeability and fouling characteristics. The collected data enabled a comprehensive characterization of the ultrafiltration pilot unit working with this particular saline feed solution, which has very high technical–economic potential.

## 1. Introduction

Sea salt production represents the oldest raw material production process in the world. This process occurs in specific shallow basins, predominantly located in the Mediterranean region, where favorable weather conditions, including wind, humidity and solar radiation, combine to facilitate continuous seawater evaporation [1]. As the chemical composition of the salt solution changes due to evaporation, various crystallization phenomena may occur, thus resulting in the precipitation of less soluble salts, including CaCO_3_ and CaSO_4_ [2]. Typically, once these two salts are extracted, the saline solution is saturated in NaCl in the last ponds, and its precipitation begins. The resulting salt solution, known as bittern, still saturated in NaCl, is considered a waste and is commonly discarded into the sea to end the process. Bitterns, however, are a very rich source of minerals that do not precipitate during conventional evaporation processes, thus reaching concentrations up to 50 times greater than that of seawater (see Table 1).

Thanks to the natural concentration processes of seawater, the recovery of minerals other than NaCl is an interesting option. This is the case for minerals such as Mg, K, Sr and B present in valuable concentrations or Cs, Rb, Ge, Ga, Pb and Cu, which are present in the bittern in concentrations ranging from 0.005 to 10 mg/L [6,7] (in which recovery is, therefore, more difficult and yet to be proved as being competitive with traditional mining). Many of these minerals have been classified as critical raw materials by the European Community due to their economic and strategic importance and their high supply risk. For this reason, bitterns have gained more and more interest and have been regarded as ‘alternative mines’ of valuable raw materials.

In recent years, bitterns have been increasingly used (i) as a source of magnesium for the production of magnesium hydroxide (Mg(OH)_2_) [8], magnesium carbonate (MgCO_3_) [9] or magnesium oxalate (MgC_2_O_4_) [10]; (ii) for struvite synthesis [11]; (iii) to enhance arsenate removal [12], (iv) as an absorbent medium for SO_x_, NO_x_ and CO_2_ removal [13,14], (v) as a raw material for Boron, Germanium and Gallium recovery [15,16], (vi) as a coagulant for heavy metal removal [17,18], (vii) as a neutralizing agent for civil and industrial wastewater [19,20], (viii) for fertilizer production [21] and (ix) as a source of Lithium [22,23].

As a further application, bitterns have been employed as a coagulating–flocculating agent, replacing more common agents such as FeCl_3_ or Al_2_(SO_4_)_3_ that are more expensive and healthier for human use [24]. The extremely high ionic strength can promote the destabilization of colloids. In fact, magnesium cations (Mg^2^⁺) present in bitterns neutralize the surface charges of colloidal particles suspended in water, which usually have negative charges. The addition of Mg^2^⁺ then reduces the repulsion between them, allowing them to come together and aggregate into small agglomerates that will flocculate to sediments.

The valorization of saltworks bitterns often requires preliminary purification processes, such as membrane filtration, before they can be fully exploited. Bitterns are, in fact, a very complex system in which there is a strong biological component, partially consisting of microalgae and halophilic bacteria [25]. Their presence might lead to considerable reductions in the quality of the final product.

Micro-filtration (MF) and ultrafiltration (UF) are the most widely used pretreatments for brines; they are typically adopted to guarantee the removal of suspended solids down to a size of 0.1 µm and a decrease in solution turbidity of up to 1 NTU [26]. Both families of UF membranes, ceramic and polymeric, are widely utilized in this field; however, the use of ceramic membranes could be advantageous. These, in fact, offer better chemical resistance against high chloride concentrations and are more resistant to the frequent chemical washing and backwashing required when solutions with high suspended solids content are treated [27]. An example of a real application of ceramic ultrafiltration membranes is the treatment of laundry wastewater [28] or, rather, the treatment of refinery oil in which the oil concentration can achieve a value of up to 6000 mg/L [29].

These pretreatments are often critical for downstream applications where the risk of fouling/bio-fouling and scaling can pose a serious possibility of unit damage. For example, one case of this is the use of nanofiltration (NF) membranes for the selective separation of lithium cations from solutions, which may be concentrated in the permeate stream [30].

**Figure 1:**
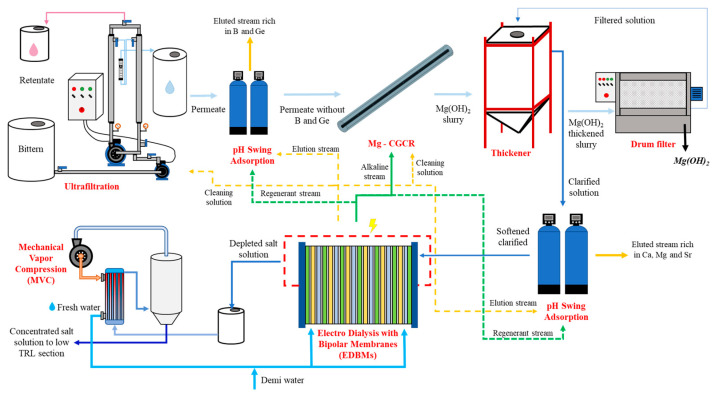
High TRL SEArcularMINE treatment chain. Solid lines represent the continuous production process. The dotted lines represent the streams of acidic (yellow) and basic (green) solutions used for the cleaning and regeneration of pHSA units.

The transition of bitterns from a waste solution to a source of valuable raw materials pushes the development of novel processes and/or technologies. The valorization chain proposed by the EU project SEArcularMINE (www.searcularmine.eu, accessed on 27 August 2024 [31]) for the exploitation of Mediterranean bitterns is a worthy example. The technical feasibility of the chain operation has been proven at the pilot scale (TRL ≥ 5) for most units by the development, construction and testing of a fully integrated pilot plant, of which the process scheme is depicted in Figure 1.

According to the process scheme, the feed bittern is first treated via ultrafiltration to remove suspended organic matter, such as microalgae, bacteria and colloids. The permeate is processed in a pH Swing Adsorption (pHSA) unit for the selective recovery of boron. After saturation, an acid-induced pH shift releases the captured mineral in the acid stream. The boron-depleted stream is sent to the magnesium crystallization reactor (Mg-CGCR), where magnesium crystallizes in the form of hydroxide. The outgoing stream is sent to thickeners, where the slurry is separated. The concentrated slurry is then sent to the drum filter to separate the solid, while the clarified stream is sent to another pHSA unit for the removal of all cations to prevent scaling phenomena in the downstream Electro-Dialysis with Bipolar Membranes (EDBMs). The latter, thanks to the water supply and an electric field, exploits the salinity of the clarified solution to produce both an acidic and basic solution in situ used throughout the chain (e.g., in the pHSA and the crystallizer), thus enhancing the circularity and the sustainability of the process. The saline stream leaving the EDBMs passes through a stage of concentration using a Mechanical Vapor Compression (MVC) unit to increase the concentration of minor ions by recovering fresh water, which is recycled back into the EDBM unit to reduce the water demand quantity. The salt solution, rich in Li^+^ and trace elements, continues its path in the low TRL treatment chain (TRL ≤4), in which all the minerals are recovered thanks to the use of selective membranes for Li^+^ and a special sorbent in other pHSA units.

The pretreatment of the bittern in this treatment chain has a significant impact on the final product. An untreated bittern would result in a significant lowering of the pHSA unit performance due to particle bed fouling and clogging, with a consequent reduction in the active surface area and decreased boron recovery efficiency. In addition, the outgoing bittern, once it enters the reactor, would lead to the formation of Mg(OH)_2_ with lower purity and, thus, lower value.

Taking this treatment chain as a case study, the influence of operating conditions and bittern properties on the performance of a UF pilot unit aimed at the removal of organic matter from real Sicilian bitterns (Trapani saltworks, Trapani, Italy) is analyzed in the present study. In addition, due to a lack of data in the literature on the rheological behavior of bitterns, a preliminary study is also proposed.

### 1.1. Description of the UF Process

Ultrafiltration is a pressure-driven process where a dispersed phase is separated from a continuous (liquid or gaseous) phase. Filtration is made possible by membranes characterized by a porous surface, with pores having a diameter in the range of 1–100 nm. The feed, once pressurized to a maximum pressure of 10 bar, is fed to the unit. A permeate stream composed of the solvent and suspended solid matter smaller than the pore size passes through the membrane and is separated from the retentate, which can be separated or recirculated back to the inlet to increase the solvent recovery. Due to its characteristics, UF is perfectly suited to food industry streams rich in suspended solids, such as those found in the milk and cheese production industries [32], in the production of fruit juices where heat sterilization would result in exponential degradation of nutrients [33,34,35,36], and also for municipal and industrial wastewaters [37,38,39].

There are several models able to predict the permeate flux $J_{v}$, one of which is the “resistance-in-series” model (see Equation (1)), which is often used for highly tortuous ceramic membranes [40].

(1) $$J_{v}=\frac{\Delta P}{\left(R_{m}+R_{f}\right)\;\mu }\;\;\;\;\left[\frac{L}{m^{2}h}\right]$$

According to this simple model, $J_{v}$ depends on four parameters: (i) the morphology of the membrane represented by the membrane resistance $R_{m}$, (ii) the permeate viscosity μ, (iii) the trans-membrane pressure ∆*P* or *TBT* representing the difference in pressures, i.e., the driving force, between the feed and permeate channels and (iv) the fouling resistance *R_f_*.

Depending on the application of UF, two different types of membranes can be employed: polymeric and ceramic. Polymeric membranes are characterized by high permeability and large membrane surfaces; conversely, ceramic membranes guarantee good chemical and thermal resistance. Other key parameters may be the performance in the presence of fouling and its reversibility. Hofs et al. [41] showed, for example, how the use of ceramic membranes enables fouling removal efficiencies higher than those pertaining to polymeric membranes, where fouling may remain adsorbed and difficult to remove even with basic washings.

In the present work, UF is devoted to pretreating the saltworks’ bitterns, which contain halophilic bacteria [42,43,44], microalgae such as Dunaliella Salina [45] and copepods and mollusks [46]. Bitterns, due to their varied chemical composition and the presence of trace elements such as nitrite–nitrogen and phosphates [47], are an ad hoc environment for the growth and proliferation of these life forms [48]. All of them have to be removed in order not to affect the final product.

In order to fully characterize the membrane behavior, it is therefore necessary to determine external factors, such as bittern viscosity and fouling potential. Unfortunately, there is a great lack of data on bittern viscosity in the literature, as this stream has been considered a waste so far. For precisely this reason, an experimental campaign was carried out to evaluate the variation in bittern viscosity as the chemical composition and temperature change.

### 1.2. Main Process Variables and Performance Indicators

In UF, the main operating variables are typically related to the feed stream. For example, as mentioned above, depending on (i) the sampling season, (ii) the evaporation and crystallization phenomena and (iii) the composition of the incoming seawater, the feed bittern exhibits different ionic compositions that strongly affect the viscosity of the solution and its organic content (depending on the concentration of the nutrients). According to the literature, the viscosity of salty solutions increases as the Mg^2+^/Na^+^ ratio and the SO_4_^2−^/Cl^−^ ratio increase [49]. The organic content is also related to the ionic composition of the bittern. As a matter of fact, bitterns represent an ad hoc environment for the growth and proliferation of particular bacterial and microalgal life forms, which might be responsible for membrane biofouling issues.

Transmembrane pressure (TMP) is the pressure required to allow the feed to pass through the membrane and reach the permeate channel. Due to pressure losses along the module, a pressure gradient takes place along the channel, and an average pressure is conventionally taken into account. The formula is as follows:

(2) $$TMP\;\left[\mathrm{bar}\right]=\frac{P_{feed}+P_{ret_{out}}}{2}-P_{perm}$$

where $P_{feed}$ is the inlet pressure at the first UF membrane corresponding to the outlet pressure of the booster pump, $P_{ret_{out}}$ is the outlet pressure at the second UF membrane read by the PI and $P_{perm}$ is the permeate pressure kept fixed at atmospheric pressure. In the presence of low fouling feed, this can be assumed to be the driving force of the process. Conversely, in the presence of high fouling feeds, such as bitterns, this assumption is not allowed as the fouling forms an additional resistance to the transport of matter, thus reducing the driving force.

Operating the unit in batch mode leads to the temperature becoming an important parameter. Without any cooling system, all the heat generated by the pumps will be dissipated inside the bitterns, thus resulting in a gradual temperature increase. Furthermore, the latter modifies the rheological properties of the bitterns by causing a rapid viscosity decrease and a consequent higher permeate flux (see Equation (1)).

To characterize the pilot, two performance indicators are employed:

- Membrane permeability “$L_{p}$”: This is a coefficient characteristic of the membrane–feed system and indicates the permeate flux through the membrane as a function of the applied pressure. The formula is as follows:
  (3) $$L_{p}\left[\frac{L}{m^{2}h\;\mathrm{bar}}\right]=\frac{J_{v}}{TMP}$$
- Organics’ Rejection “R”: This defines the amount of organic suspended matter retained by the membrane (see Equation (4)). Given the typical separation mechanism of the UF unit, only the suspended component can be removed in this process by the steric blocking mechanisms. Some of the organic matter is solubilized, but this, being within the solvent itself, cannot be retained.
  (4) $$R=\left(1-\frac{C_{org_{per}}\;}{C_{org_{feed}}\;}\right)*100\;\;\;$$
  where $C_{org_{per}}$ and $C_{org_{feed}}$ are the organic compound concentrations (mg/L, measured via Chemical Oxygen Demand (COD) analysis) present in the permeate volume and feed volume, respectively.

In order to ideally maximize membrane efficiency, a membrane with high permeability and high rejection would be required. This is often physically impossible as high permeabilities are provided by looser membranes exhibiting lower rejections (due to the higher passage of suspended material through their larger holes).

Many organic compounds are quite soluble in water, even at concentrations around mg/L. These include aromatic benzene and its derivatives (xylene and butylbenzene), linear and cyclic alkenes (n-pentane and n-hexane), etc. The literature shows a decrease in the solubility of these compounds as the salinity of the solution increases due to the “salting-out” thermodynamic phenomenon. In further detail, organic compounds are quite soluble in water thanks to the hydrogen bonds that create a solvation shell. As the amount of salt increases, the competition between dissolved “charged” ions and “uncharged” organic molecules increases as well. The higher the salinity, the fewer water molecules are available to solvate the organic molecules, which, no longer solvated, start to interact with each other, forming larger aggregates until they precipitate. Xie et al. [50] showed how the solubility of some organic compounds decreases by up to 60% in seawater, with a salinity of 3.5% *w*/*w* compared to distilled water.

Clearly, this effect is exacerbated in high-salinity solutions like brine and even more in bitterns. Thus, given the very low solubility of these compounds, rejection can be considered an index of the removal of suspended organic matter in solution, thus assuming that the organics dissolved in the bittern (organic acids such as lactic acid or acetic acid and alcohols produced by the aerobic and anaerobic respiration of microorganisms in the bittern) are negligible.

### 1.3. Description of the UF Pilot Unit and Experimental Procedures

**Figure 2:**
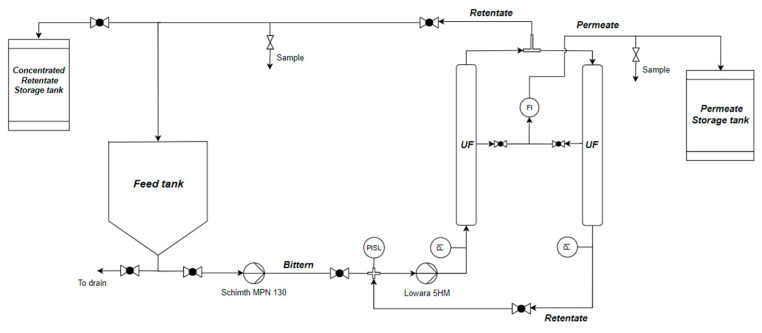
Piping and instrumentation diagram of the UF pilot plant.

The ultrafiltration pilot unit was customized for the treatment of concentrated brines and was supplied by Sepra SrL (Italy). The pilot is composed of two identical membrane vessels, each equipped with two asymmetric ceramic membranes (CéRAM^TM^—TAMIS INDUSTRIES, OD 25 mm and L = 1178 mm, 23 channels) with a total area of 0.7 m^2^ installed. The membrane has a molecular weight cut-off of 300 KDa and a water permeability (measured with pure water at 25 °C) of 650 L/(m^2^hbar) (internal communication of the authors with Sepra SrL). The TiO_2_ support and ZrO_2_ as an active layer make the membrane resistant to pH values ranging from 0 to 14, maximum temperatures of 250 °C and a maximum pressure of 10 bar. The plant is equipped with two variable-speed feed pumps: a load pump (Schimth MPN 130) and a booster pump (Lowara 5HM). An external power supply is used to provide the voltage to the load pump in order to tune the operating pressure of the unit. Pressure monitoring was performed using two gauges (i.e., pressure indicators (PIs) in Figure 2) while the flowrate was monitored using a rotameter (FI in the figure).

The hydraulic circuit makes it possible to operate the system either continuously, using the two membranes either in series or in parallel but obtaining small recovery percentages (<5%), or in batch mode by recirculating part of the retentate within the first module and the other part within the feed tank, thus guaranteeing, over time, reaching permeate recovery values >95%.

The tests were conducted in batch mode using an initial feed bittern volume of 100 L. All tests were stopped when the permeate recovery was higher than 90 (i.e., final feed bittern volume < 10 L). At the end of each test, the cleaning procedure was carried out by flushing with demi water to perform a bittern displacement and then with an alkaline solution (pH 13) to remove organic fouling deposited on the membrane.

Also, one long-run test was carried out to confirm the reliability of the plant over time. A refilling strategy was adopted for the long run operation: the test started with a feed volume of 150 L, and once the feed volume in the storage tank reached a minimum value of 50 L, the tank was filled up to 150 L with fresh feed to restore the initial volume, allowing for the continuous operation of the plant. In addition to this, when a minimum flux value of (20 L/m^2^h) was reached and no increase in temperature was encountered, the membrane cleaning procedure was implemented to restore the initial flux.

**Figure 3:**
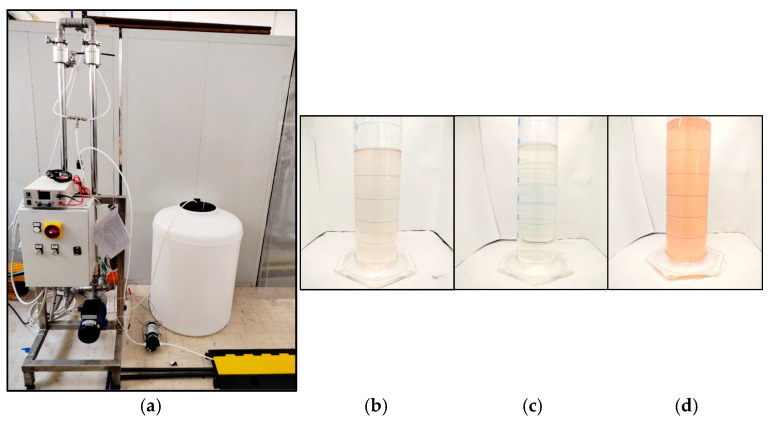
(**a**) A picture of the ultrafiltration pilot unit installed in the SEArcularMINE treatment chain; (**b**) bittern, (**c**) permeate and (**d**) retentate samples.

During the tests, data regarding the permeate flow rate, temperature, conductivity of permeate (Figure 3c) and retentate (Figure 3d) and pressure were periodically collected.

The behavior of the pilot unit was studied by investigating three ‘case studies (CSs)’ distinguished by a variation in the input feed, as shown in Table 2. The three case studies deal with different bitterns, taken from the Trapani saltworks in Italy, during three different seasons: July 2023 (CS-1), October 2023 (CS-2) and March 2024 (CS-3). As the season changes, the chemical composition of the brine changes as well, depending on the different evaporation and crystallization rates. Similarly, the organic composition (living and non-living) changes as well due to its close relationship with the salinity of the solution.

## 2. Materials and Methods

Flow measurements were obtained using a glass 500 mL graduated cylinder and a timer. Temperature, conductivity and pH were monitored during the tests using a WTW™ pH/Cond 3320 Universal Multi-parameter Portable Meter in both the retentate and permeate flows. The pH probe was calibrated using a 4–7–10 standard pH buffer solution, while the conductivity probe (WTW TetraCon^®^ 325) was calibrated using a 1413 μS/cm standard solution.

Ion chromatography was used for cation and anion characterization. For cation analysis, a Metrohm 882 compact IC plus column was used with 0.5 mM of oxalic acid and 4.5 mM of nitric acid solution as the mobile phase. For anion analysis, the mobile phase was a Na_2_CO_3_ 3.2 mM—NaHCO_3_ 1 mM solution used with the Methohm 930 compact IC plus column. The samples were filtered through 0.45 µm Nylon Filter-lab filters and diluted in ultrapure water. In addition, an inductively coupled plasma atomic emission spectrometer (ICP-OES, Optima 2100 DV, PerkinElmer, Waltham, MA, USA) was used for the detection of boron. Bittern samples were diluted in 2% *w*/*w* nitric acid before measurement.

Chemical Oxygen Demand (COD) was carried out using a Spectroquant^®^ cuvette test (1.17058) and MQuant^®^ Chloride Test (1.11132) provided by Merck. Due to the high chloride concentration, a chloride removal step was carried out according to the DIN 38409-41-2 [51] procedure. To determine the COD value, a calibration curve was created using increasing COD solutions, in the range between 0 and 60 mg/L, of glucose (Glucose, D(+) 99+%) provided by Chem Lab. The calibration line (72.442−294.74∗Absorbance, $R^{2}=0.997$) was obtained using a spectrophotometer (Agilent Technologies Cary 60 Uv-Vis) at a wavelength of 444 nm. To be within the reliability range of the kit, the bittern samples were diluted in ultrapure water.

Viscosity data were obtained using a Cannon-Fenske 25-Series (0.4–1.6 Cst) and a Cannon-Fenske 75-Series (1.6–6.4 Cst) capillary viscometer provided by Levanchimica Srl. Viscosity data at increasing temperatures were obtained using a temperature-controlled bath.

Density tests were performed by weighing 50 mL of fluid in a flask on a precision balance. Density data at different temperatures were obtained by preheating the sample in an oven (TCN 50 Plus) to the set temperature and weighing the sample by adding or subtracting volume until a fixed flask volume of 50 mL was reached.

## 3. Results

### 3.1. Analysis of the UF Operation in Terms of Solution Inorganic Composition and Density

**Figure 4:**
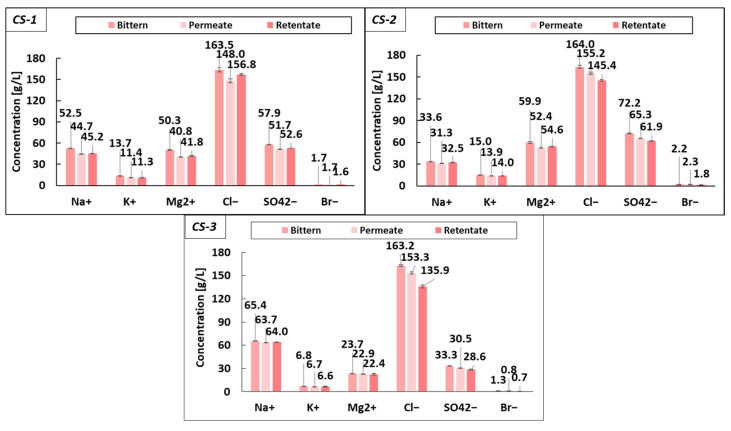
Comparison of permeate, retentate and feed bittern composition (operating UF pilot at 3 bar) for the three case studies.

The analysis performed on the composition of permeates and retentates compared with the feed bittern showed a variation in the salt composition of the streams. Specifically, relevant data corresponding to the operating pressure of 3 bar are reported in Figure 4 for the three case studies.

In Case Study 1, the concentration of monovalent cations in the permeate decreased by approximately 15%, while the concentration of Mg^2+^ showed a reduction of about 19%. Following the same trend, the chloride ion (Cl^−^) concentration decreased by ~9.5%, while sulfate ions (SO_4_^2−^) showed a reduction of ~10.7%. The same behavior was found in CS-2, in which the retention of monovalent cations was 7.3%, the Mg^2+^ rejection was 12.6% and the anion rejection was 5.4% and 9.6% for chloride and sulfate, respectively. In Case Study 3, the sodium and potassium ion concentration in the permeate was almost unchanged (2% reduction), while the Mg^2+^ concentration showed a minor reduction of 3.4%. The chloride and sulfate ions exhibited decreases of 6.0% and 8.6%, respectively.

Such a reduction in saline content in both the permeate and retentate can likely be attributed to salt absorption phenomena in the fouling layer generated on the membrane surface, as demonstrated by the high conductivity observed in the washing water after washing cycles. However, a more in-depth investigation is needed in order to better identify and quantify such phenomena.

**Figure 5:**
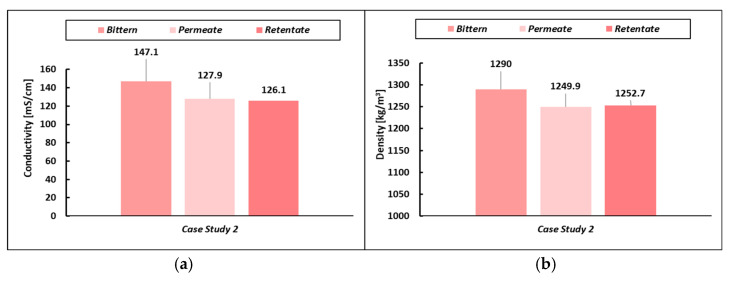
(**a**) Conductivity and (**b**) density variation in permeate and retentate stream compared with the bittern feed for the CS-2.

The small reduction in the saline contents in the permeate and retentate streams can be further confirmed by the reduction in density and conductivity observed in the outlet streams compared to the feed solution (see Figure 5).

**Figure 6:**
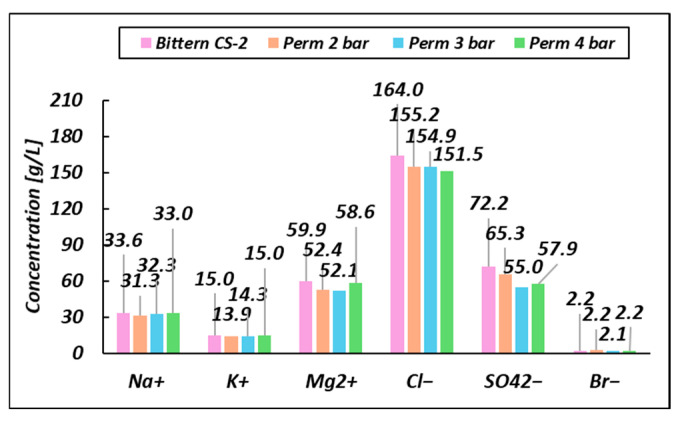
Comparison of CS-2 permeates (and original feed bittern) composition as a function of the inlet pressure ($P_{feed})$.

A further experimental analysis was performed for the CS-2 case by operating the UF pilot at different applied pressures in order to assess the effect of this operating parameter on the separation performance. The ion concentrations observed under different pressures are summarized in Figure 6.

### 3.2. Viscosity of Saltworks’ Bitterns

**Figure 7:**
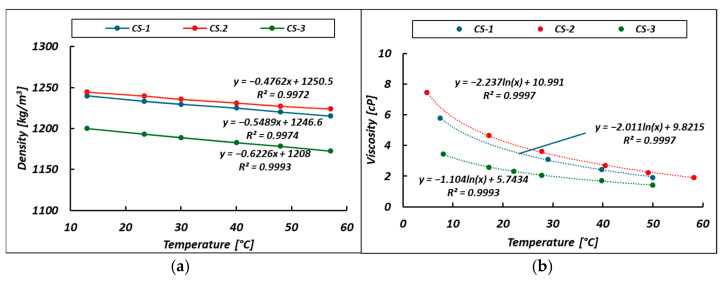
Bittern permeate density (**a**) and viscosity (**b**) as a function of temperature. Points represent the experimental data. Curves indicate linear (**a**) and logarithmic (**b**) interpolation laws.

To characterize the behavior of the unit, a viscosity study of the permeated bittern was conducted using a capillary viscometer with a thermostatic bath. The cinematic viscosity (ν) was converted into a dynamic viscosity (μ), according to the usual equation μ=ν∗ρ. For this purpose, the density variation with the temperature was also included in this study. The density points reported in Figure 7a are well fit by a linear relationship $\rho =a_{0}+b_{0}\;T\left[{}^{\circ}C\right]$. The viscosity versus temperature experimental data shown in Figure 7b are well fit by logarithmic curves $\mu =a+b\;ln\left(T\left[{}^{\circ}C\right]\right)$. All the corresponding coefficients and *R*^2^ are reported in Figure 7.

Efforts are presented in the following section to propose a new yet simple correlation for the estimation of bittern viscosity on the basis of its magnesium ion concentration.

Following the structure of the two rheological models (Equations (8) and (9)) available in the literature for artificial solutions presented in the following section (Section 4.2), a new model was developed on the basis of the experimental viscosity measurements carried out in the present work for real permeated bitterns. Compared to the literature equations, the proposed one (Equation (5)) has two main differences:

- The Mg^2+^ molar concentration was preferred as a representative parameter of the bittern salinity;
- An additional addendum was added in order to allow the equation to follow the variation in the viscosity with the temperature.

(5) $$\frac{\mu }{{\left.\mu_{wat}\right|}_{T_{\left[{}^{\circ}C\right]}}}=1+A_{1}\sqrt{C_{Mg}\left[M\right]}+A_{2}C_{Mg}\left[M\right]+A_{3}{\left(C_{Mg}\left[M\right]\right)}^{2}+A_{4}C_{Mg}\left[M\right]\ln\left(T\left[{}^{\circ}C\right]\right)$$

**Figure 8:**
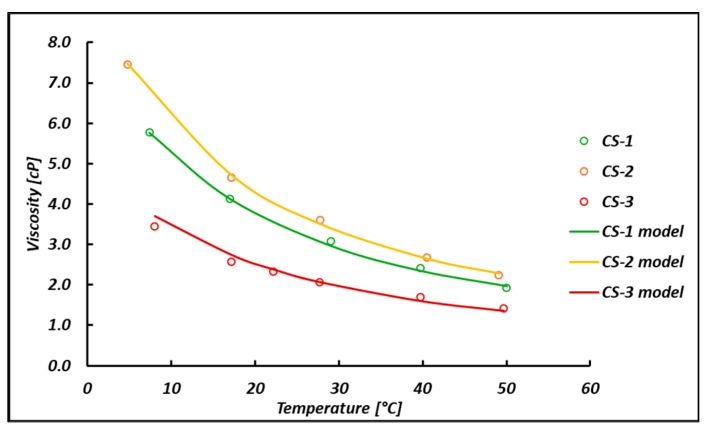
Comparison between experimental (symbols) and predicted (curves) viscosity as a function of the temperature.

The model was calibrated by means of the experimental data collected in the present work for the case of the real permeated bittern CS-1, CS-2, CS-3 and using seawater viscosity data [52]. These data include the variation in viscosity as a function of Mg^2+^ ion concentration and temperature (Figure 8). The constant parameters resulting from the calibration are reported in Table 3 and are such as to obtain an MSE (Mean Squared Error) value of 0.0036. It is worth mentioning that no appreciable change in solution viscosity with pressure is expected. In fact, as shown by Francke and Thorade [53], the viscosity/pressure trend for brines up to 5 mol/kg is almost constant, up to 500 bar. Therefore, for the pilot plant operating range, where the maximum allowable pressure is 10 bar, the influence of pressure can be considered null.

**Figure 9:**
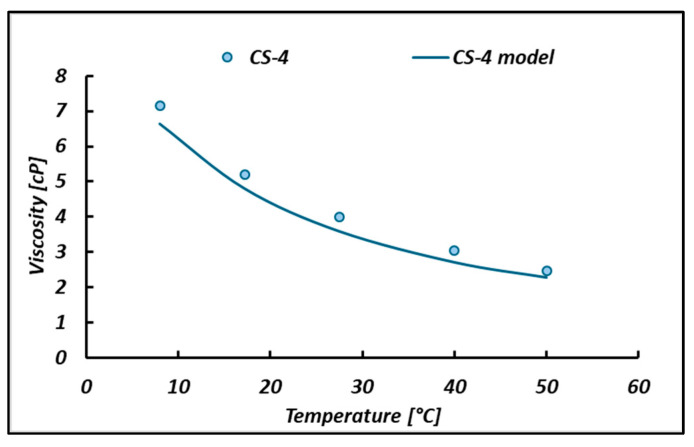
Prediction of May 2024 bittern (CS-4) viscosity.

Once the rheological equation was calibrated, it was validated by predicting purposely collected experimental data and literature data. More precisely, the model was first validated by trying to predict the viscosity of a different permeated bittern sampled and filtered in May 2024, exhibiting a Mg^2+^ concentration of 2.37 M, here known as the CS-4 bittern (Figure 9). Good agreement can be seen with an average relative error of 7%.

**Figure 10:**
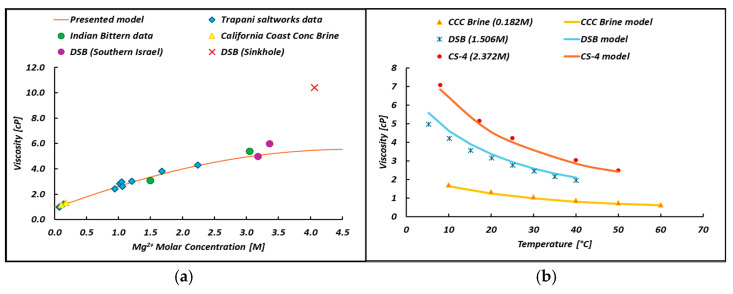
(**a**) Comparison between real (symbols) and predicted (curves) data of different bitterns at 20 °C; (**b**) comparison between real (symbols) and predicted (curves) data for the viscosity–temperature point present in the literature.

The model capability was also tested for the case of (i) other real bitterns of known viscosity (samples from the Trapani saltworks, in the period of May–June 2023; blue square [54]), (ii) on real Indian bittern viscosity data (B1–B2; green circles points) [55], (iii) concentrated seawater solution from the California coast (CCC) (7% and 12% of salinity; yellow triangles) [56], (iv) Dead Sea brines [57,58] and (v) Sinkhole brines from the Dead Sea. All the data reported in Figure 10a can be found in the literature. Importantly, the model’s capability of fitting the viscosity variation with temperature was also tested for the case of bitterns of a different kind [57] (see Figure 10b). More precisely, Dead Sea Brine (DSB) bittern data and concentrated seawater brine (12% salinity) from the California coast were adopted for comparison.

### 3.3. Permeability Data and Trends

**Figure 11:**
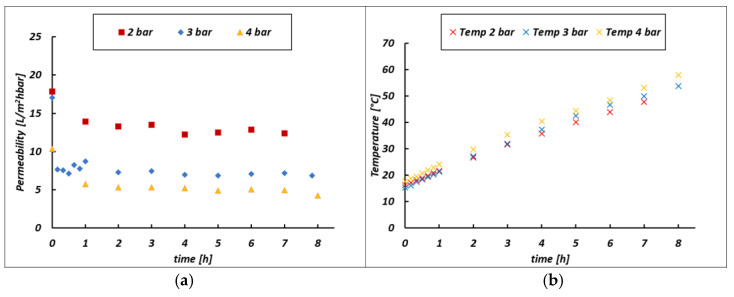
(**a**) Membrane permeability and (**b**) temperature trends versus time at variable inlet pressures ($P_{feed})$ for the case of the CS-2 bittern.

**Figure 12:**
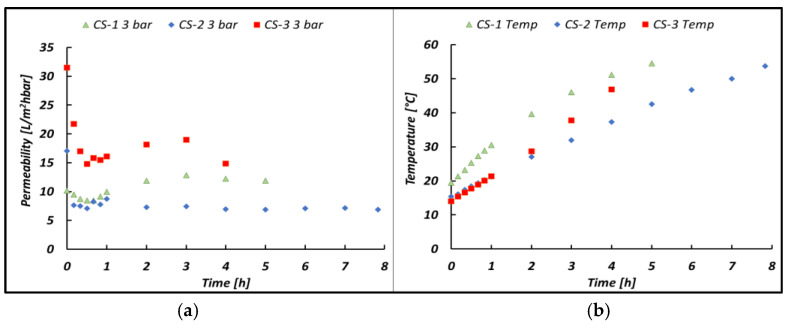
(**a**) Membrane permeability and (**b**) the temperature increasing trend versus time when varying the feed bitterns processed in the UF pilot at 3 bar inlet pressure ($P_{feed}$). The CS-1 test was conducted with a batch volume of 50 L.

Figure 11 shows the change in permeability when a higher pressure was applied as a driving force. This drop is 13, 7.4 and 5.2 L/m^2^hbar when applying 2, 3 and 4 bar, respectively, as the inlet pressure ($P_{feed}$) or 1.6, 2.5 and 3.2 as a TMP. This behavior can be explained thanks to an increase in the fouling resistance, as discussed in the following section. With reference to Equations (1) and (3), an increase in operating pressure should lead to an increase in permeability, which has not yet been recorded due to a corresponding increase in fouling resistance, which has a predominant effect. Figure 12 shows how permeability changes when a different bittern is fed to the UF unit.

### 3.4. Fouling Resistance Characterization

The membrane resistance $R_{m}$ was derived from the membrane technical datasheet. This, in fact, can be calculated as shown in Equation (6):

(6) $$R_{m}=\frac{\Delta P}{J_{v_{wat}}\;\mu_{wat}}-R_{f}\;\;\;\;\to \;\;R_{m}=\frac{\Delta P}{J_{v_{wat}}\;\mu_{wat}}\;\;\;\to \;\;R_{m}=\frac{1}{L_{p_{wat}}\;\mu_{wat}}\;\;\left[m^{-1}\right]$$

At room temperature, in treating distilled water as a feed (assuming the absence of fouling resistance), the membrane resistance $R_{m}\;$ has a value of $6.22\times 10^{11}\;\left[m^{-1}\right]$, representing a value close to the others found in the literature [59].

The characterization of fouling within the membranes was obtained empirically by obtaining information on $R_{f}$ from the flux data recorded during the tests. More precisely, once the membrane resistance $R_{m}$ has been derived (see Equation (6)), and the viscosity trend has been determined by experimental tests, *R_f_* can be derived from the Equation (1) as follows:

(7) $$R_{f}\left[m^{-1}\right]\;\;=\frac{\Delta P}{J_{v}\;\mu }-R_{m}$$

**Figure 13:**
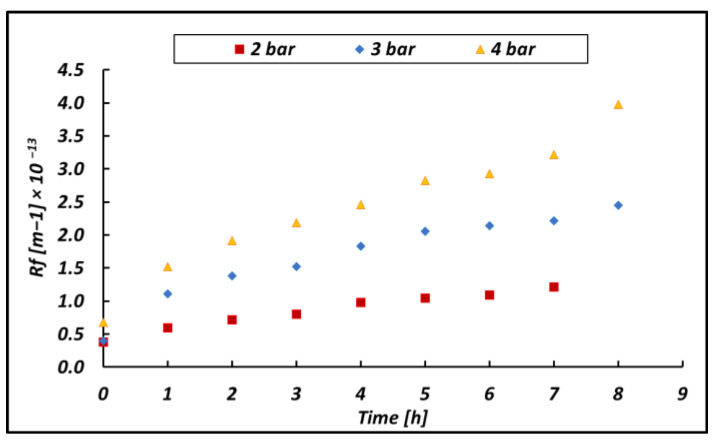
Fouling resistance versus time as a function of the inlet pressure ($P_{feed})$ for the CS-2 bittern.

The variation of $R_{f}$ with time as a function of the operating pressure is shown in Figure 13.

**Figure 14:**
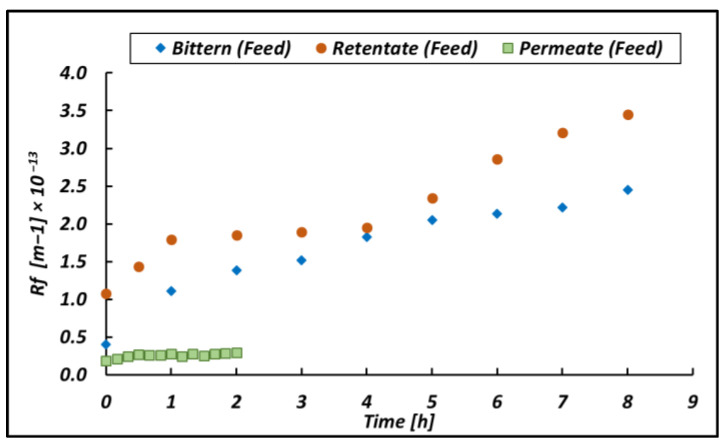
R_fouling_ versus time in the case of feeding the UF unit with the original CS-2 bittern; the permeate produced in a previous CS-2 test; and the retentate produced in a previous CS-2 test. The UF pilot was operated at 3 bar as a $P_{feed}$.

Similar and additional tests were also conducted by feeding the retentate and the permeate obtained by ultrafiltering the CS-2 bittern to the UF pilot. In other words, the permeate and the retentate obtained from the first UF test were used as new feeds for a second and third test; relevant results were compared with those relevant to the original CS-2 bittern and reported in Figure 14.

### 3.5. Experimental Results on Organic Rejection

**Figure 15:**
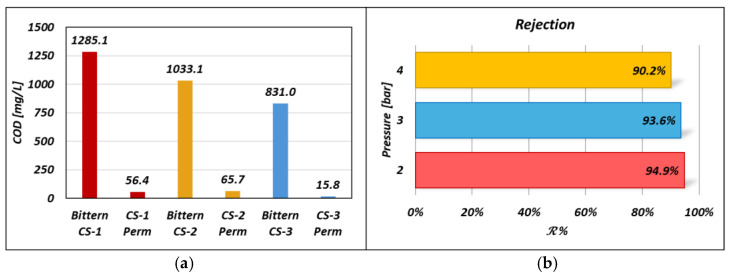
(**a**) Bittern and permeate COD for the three different test cases while operating the UF unit at 3 bar as the inlet pressure ($P_{feed}$). (**b**) Organic rejection as a function of the inlet pressure ($P_{feed}$) for the case of the CS-2 bittern.

The organic rejection performance of the UF pilot was tested for the case of the three bitterns while operating the unit at 3 bar. Relevant results are reported in Figure 15. Organic rejection R data are obtained by COD analyses performed for both bittern and permeate streams. Notably, for all the three test cases, the R values were higher than 90%; in particular, CS-1 had a rejection rate of 95.6%, while CS-2 rejection was 93.6% and CS-3 was 98.1%. With this in mind, once the first gel layer of organic matter is created, the organic matter that continuously enters the system is allegedly easily adsorbed onto it, contributing to the growth of $R_{f}$. These values are also consistent with the application of a similar membrane (cut-off value of 150 kDa) to the dairy wastewater treatment, in which a COD removal efficiency value of 95 ± 1% was found [60].

The influence of the applied pressure on the organic rejection was investigated; in particular, Figure 15b shows the rejection R versus the pressure P trend for the case of the CS-2 feed bittern. Also, in this case, the rejection coefficient is above 90%, but a rejection–pressure trend of the rejection was found.

### 3.6. Long Run Test: Pilot Performances

**Figure 16:**
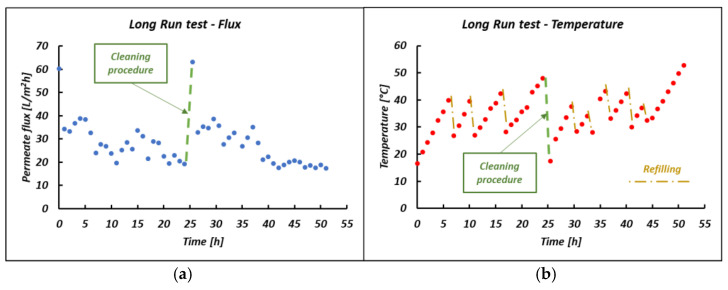
(**a**) Membrane flux and (**b**) temperature trend over time in the long run test.

**Figure 17:**
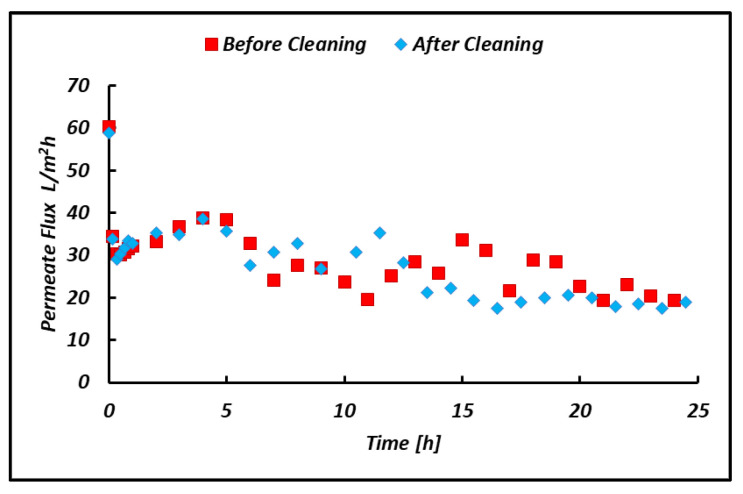
Comparison of the initial membrane flux and the restored membrane flux after a cleaning procedure.

A long run test was performed for more than 50 h of continuous operation, with an intermediate alkaline washing step undertaken to remove organic fouling and restore the initial permeability of the module. The long run test was carried out by treating the CS-3 bittern under a fixed TMP of 2 bar. Interesting results are reported in Figure 16 and Figure 17, in which the flux behavior was investigated. As shown in Figure 16a, the permeate flux trend was quite stable over time following the temperature trend (see Figure 16b) until the fouling became predominant and the test was interrupted for the cleaning procedure. Once the membrane’s original status was restored, the transmembrane flux was found to perfectly overlap with the one found initially. This behavior, shown in Figure 17, highlights the presence of superficial reversible fouling, easily removed by flushing with an alkaline solution.

## 4. Discussion

### 4.1. Inorganic Composition Variation

Although the separation mechanism of a UF unit does not involve the separation of ions, a non-negligible retention of ions in all three cases studied was found. Several studies in the literature show similar behaviors. For example, Guo et al. [61], using an Amicon S10N1 ultrafiltration cartridge with 1 kDa spiral winding to ultrafiltrate low-salinity water (up to 1.95 *w*/*w*%), obtained similar ion rejection rates resulting in a decrease in their quantity in the permeate. All major cations show a steady decrease in their retention coefficients with increasing salinity, with values around 10% at higher salinity points.

More recently, Xing et al. [62] showed the rejection of Na^+^ (1.5% ± 10.6%), K^+^ (0.5%± 10.7%) and Mg^2+^ (4.6% ± 10.3%) ultrafiltrating seawater (Mediterranean Sea) using a 1–8 kDa ceramic ultrafiltration membrane with a TiO_2_/ZrO_2_ active layer. The above rejection values may change depending on the operating pH of the unit, which if found to be greater than the IsoElectric Point (IEP) of the membranes (≈5.5) is likely to negatively charge the surface. Since the membrane is negatively charged, there could be rejection for anions due to electrostatic exclusion, while there could be a higher retention of cations due to electrostatic attraction phenomena.

Surprisingly, no data on the final composition of the retentate are available in the literature. As can be seen in Figure 3, a reduction greater than that expected was found for the ion concentration in the retentate, thus leading mass balances to appear unclosed. One possible cause of this could be attributed to the fouling present within the unit (see Section 3.4). Since the membrane is negatively charged, there is an accumulation of cations on the membrane surface, and these could be trapped within the ever-growing layer of fouling formed during the filtration process, as mentioned in the following section. This is further confirmed by experimental evidence for which the decreasing trend in ion retention follows the decreasing trend in the amount of organic matter in the solution (see Table 2, COD). In addition to this, the colloids on the surface, with a negative charge, increase the cation retention effect. It has also been shown that the accumulated fouling layer can create microenvironments on the membrane surface that affect the local distribution of ions, leading to non-uniform retention [63].

As shown in Figure 6, as the pressure increases, more and more cations are able to pass through, also aided by attractive force due to the negative superficial charge of the membrane. For the same reason, there is an increase in repulsive forces between superficial and anions as pressure increases, and this is reflected in a decrease in ion flux. This trend suggests that the selectivity of the membrane is influenced by both the nature of the ions and the UF operational parameters.

### 4.2. Bittern Viscosity

An in-depth comparison of the viscosity with the ionic composition data of the permeated bitterns shows that the higher the Mg^2+^/Na^+^ ratio, the higher the bittern viscosity [58]. Interestingly, temperature dependence is also related to chemical composition and, in particular, seems to depend on the molar concentration of magnesium in the permeate stream. A similar trend can be found in the literature for the case of the Dead Seas Brine (DSB) [57] (with a Mg^2+^ concentration equal to 36.6 g/L, corresponding to 1.506 M) in which the viscosity–temperature trend of this bittern can be fit with the function $\mu =7.6265-1.518\;\ln\left(T\left[{}^{\circ}C\right]\right)$ with a $R^{2}=0.993$.

For the determination of the viscosity of electrolyte solutions, the Jones–Dole law (Equation (8)) is often used [64].

(8) $$\frac{\mu }{\mu_{wat}}=1+b*\sqrt{C}+d*C\;$$

where *b* is a coefficient that describes the impact of charge–charge interactions on the viscosity of a solution, d is a coefficient that characterizes the solute–solvent interactions at a defined temperature and pressure and C is the solute concentration. This law works well for solute concentrations lower than 1 M because the viscosity of highly concentrated solutions increases exponentially until it diverges for a critical concentration. Qiblaway et al. [49] also propose an extended Jones–Dole’s equation, valid for artificial NaCl—MgCl_2_ solutions at higher concentrations (see Equation (9)).

(9) $$\frac{\mu }{\mu_{wat}}=1+A_{1}\left(\sqrt{C_{1}}+\sqrt{C_{2}}\right)+A_{2}\left(C_{1}+C_{2}\right)+A_{3}\left(C_{1}^{2}+C_{2}^{2}\right)$$

Both formulations, calibrated and tested for an artificial solution, are based on the mass concentrations of the salts present in the solution. This requires the estimation of the mass concentrations of the salts from an analysis of the ionic composition of the solution. This, when applied to real solutions of unknown salt concentrations, adds more complexity because of the prediction of the different salts formed from a simple ion analysis.

Some efforts were carried out in the literature to predict the brine viscosity to better model and design desalination units, such as Multiple Effect Distillation (MED) or Multiple Stage Flash (MSF) distillation. In this regard, several models are found in the literature, but all of them take into account the salinity of the brine in a small range (up to 100 g/L) [65]. The common model for the brine viscosity is reported in Equation (10).

(10) $$\mu =\mu_{w}\left(1+Q_{1}S+Q_{2}S^{2}\right)$$

where $Q_{1}$ and $Q_{2}$ are constant but a function of temperature, and *S* is the brine salinity expressed in terms of the g/1000 g saturated solution.

The presented model (Equation (5)) predicts the bittern viscosities over a concentration up to 2.5 M in terms of Mg^2+^ molar concentration. Within this range, the error with the experimental measurements falls within ±5%, while when the molar concentration of Mg^2+^ exceeds 3 M, the error committed increases to 15%. The error committed within the calibration range of the presented model is very close to the error reported in the common brine viscosity models (Equation (8)), making the presented model reliable.

At higher concentrations than reported, bitterns become a complex system characterized by the presence of microcrystals of suspended salts. The presence of solids has an effect on viscosity. For example, the addition of 4.8% and 17% fine KCl (with an average diameter of 130 μm) raised the brine’s viscosity from 6 cp to 9 and 18 cp, respectively [58]. Although there are no studies on the rheology of salt brines, it is generally assumed that nonpolymeric low-molecular-weight solutions behave as Newtonian fluids [66]. In the latter stages of evaporation, when concentrations are high, and the number of nuclei and crystals in suspension becomes appreciable, deviations from ideal Newtonian behavior may occur. This would explain the high viscosity (10.4 cP) recorded of the Sinkhole Brine [57] (4.1 M Mg^2+^). To confirm, the highest viscosity (22.8 cP) was found in the last ponds of Sfax’s saltworks [67], in which the molar concentration of magnesium exceeded 5 M. This suggests that the present simplified model should not be used for magnesium concentrations in bitterns beyond the limit validity value (i.e., 2.5 M Mg^2+^) for which the model was calibrated.

Regarding the temperature variation in the viscosity, very good agreement was found (with an average prediction error of 7%), thus confirming the reliability of the proposed rheological equation in predicting the viscosity of bitterns of different origins even at different temperatures.

Overall, notwithstanding the model’s attempts to correlate the viscosity, which is strongly dependent on all ions in the solution, to the presence of the Mg^2+^ ion alone, it is able to provide good predictions for a number of cases.

### 4.3. Membrane Permeability

Usually, in a batch ultrafiltration test, the permeability trend should decrease over time due to the increase in fouling resistance. However, as shown in Figure 11, this is not the case (i.e., a constant trend is shown), and this is due to the temperature variation. As a matter of fact, the UF pilot is operated in batch mode, and the temperature increases with time (see Figure 11b) due to the heat transferred by the pumps to the solution streams during the recirculation. As described in the previous section, a temperature increase yields a logarithmic reduction in the solution viscosity, which, in turn, can lead the permeability to increase according to Equations (1) and (3). Basically, starting with the first hour, there are two counteracting effects (i.e., $R_{f}$ increase and viscosity decrease over time), which somehow compensate for each other, as confirmed by the slightly variable permeability versus time trend reported in Figure 11a. During the first hour of operation, the predominant effect is the growth of the fouling resistance, which is caused by the accumulation of the suspended solid along the membrane surface. As they are larger than the membrane pores, they are rejected by the membrane, forming a gel layer that is considered the cause of the increasing fouling resistance and the initial drop in the bittern permeability. The same behavior was also found when dairy wastewater, very rich in suspended solids, was treated with ceramic membranes [60].

Moreover, permeability is expected to reduce when the membranes are further covered by fouling (i.e., higher $R_{f}$) and this is somehow confirmed by Figure 12a; in accordance with Table 2, CS-3 is the bittern exhibiting the lowest COD and, as a consequence, we should expect lower fouling and the highest permeabilities. The opposite occurs for the CS-2 bittern, which was found to be the most viscous in Section 3.2 and the most foulant, with the highest COD. In further detail, in the CS-2 case, the combination of higher viscosity and higher organic matter content resulted in a lower average permeability value (7.4 L/m^2^hbar) compared with the CS-1 case (10.3 L/m^2^hbar), while the low viscosity and foulant properties of CS-3 at the beginning of the test yielded a higher permeability (17 L/m^2^hbar), leading to a shorter treatment time for this type of bittern.

### 4.4. Fouling Resistance

As expected, the fouling resistance $R_{f}$, increases with time due to the accumulation phenomena of organic matter on the membrane surface. As shown in Figure 13, the applied pressure plays a crucial role in determining the fouling resistance: the higher the pressure, the fast the fouling growth. This behavior is allegedly caused by the increased pressure exerting a cohesive force on the existing organic substrate matter attached to the membrane. A higher fouling resistance led to a lower flux through the membrane, and the final result was an increase in the operational time to treat the batch volume. In addition, Figure 13 also shows also an increase in the initial fouling resistance obtained at the beginning of the test. This may be due to an increasing initial thickness of the gel layer promoted by the increased pressure.

In order to simulate different scenarios with different foulant properties of the bittern, the permeated and retentated bittern of the CS-2 were also ultrafiltrated.

As shown in Figure 14, *R_f_* relevant to the permeate feed is quite low, as expected, because most suspended matter has already been removed during the first UF step. However, a small increase of *R_f_* with time is observable in Figure 14 due to fouling phenomena caused by the low concentration of suspended solids present in the permeate due to the lack of full rejection of the UF membrane. One order of magnitude lower fouling resistance led to a very short (i.e., 4 times shorter) filtration time due to a strong increase in the permeate flux.

On the other hand, during the ultrafiltration of the retentate, higher initial R_f_ values were observed, but the growth rate of the fouling was similar to the one found when treating fresh bittern. Due to the high fouling level of the membrane, at the end of the 8th h, only a recovery of 70% was obtained, a value clearly lower than the 95% recovery obtained at the same time by processing fresh bitterns. Interestingly, the high organic content of the feed has a clear relevance at the beginning of the test ($R_{f_{0}}$), where the fouling barrier is formed but seems to have no clear relevance in the growth of fouling resistance; this is comparable with a bittern 10 times less concentrated.

In summary, the low fouling resistance of the permeate, 10 times lower than in the case of fresh bittern, confirms the suspended solids’ removal effectiveness of UF, clearly indicating that this technology is a promising pretreatment for this unconventional type of feed. Moreover, even when the permeate bittern was processed in the UF unit, the fouling resistance still has a not-negligible value, 1 order of magnitude higher than those found when treating river water (such as the case of the Odra river) [68]. This permeate bittern is a hypersaline solution and might be attributed to the presence of scaling on the membrane surface. The high concentration of various ions, the variation in temperature and the possible interaction with the membrane surface could lead to complex precipitation dynamics.

### 4.5. Organic Rejection

As shown in Table 2, the COD values found in the feed bittern samples are quite different from each other as they are consistent with the growth seasonality of bacterial and microalgal colonies typical of seawater bitterns. In fact, these halophilic life forms tend to develop contextually to evaporative processes. Here, the maximum organic content was found in CS-1 (July 2023); there was a lower value in CS-2 (October 2023), where the colonies begin to suffer seasonality; and the lowest one was found in CS-3 (March 2024) as it was sampled at the end of the cold season of the evaporating basins.

It is worth focusing on the pressure effect: the higher the pressure, the worse the UF unit performance in terms of COD removal, as shown in Figure 15b. More precisely, lower performance was found when shifting from 2 to 3 bar, while a sharper decrease was obtained at 4 bar. Clearly, as the operating pressures increase, the mechanical stress exerted on the organic component increases as well. In particular, the higher the stress, the higher the rate of destroyed cells, thereby leading to an increasing trend of organic matter composed of cellular debris and intracellular organic content (expelled by the cell during its explosion). This smaller organic matter, in addition to an increased feed-side pushing force, results in an increasingly pushed passage of organic matter towards the permeate, thus reducing the quality of the final product.

Although the higher pressure leads to higher debris production, the accumulation of organic matter on the membrane surface helps to limit the reduction in rejection efficiency. Due to fouling, the rejection of suspended solids and debris increases because these, even smaller than the pore size, are retained by a compact fouling layer [60].

### 4.6. Long Run Test

A 52 h long run test (see Figure 16) was also performed to assess the stability of the system. Again, during the first few hours, a balance was found between the continuously increasing fouling resistance in the system and the solution viscosity reduction due to temperature variations. Precisely because of the temperature variation in the system, due to the continuous refills of the fresh solution, it was not possible to obtain a constant flow trend as shown in precedence. However, since the temperature trends should be overlapped, the huge influence of temperature in the ultrafiltration process is again confirmed. It is worth mentioning that the balance between fouling and viscosity tends to decrease over time, showing a predominant effect of fouling. In fact, despite the increase in temperature, the permeate flux steadily decreased.

As regards Figure 17, the initial flux was restored after the cleaning procedure. This similar trend should indicate the presence of reversible fouling, considering the negligible presence of irreversible fouling.

## 5. Conclusions

An ultrafiltration pilot plant was tested as part of an integrated treatment chain for the production of minerals from seawater bitterns in the framework of the activities of the European Horizon 2020 SEArcularMINE project. The plant was installed and operated to treat real bitterns collected from the local saltworks in Trapani (Sicily, Italy) and sampled during different seasons.

The pilot plant targets a very high recovery in permeate, minimizing the retentate volume, fully rich in suspended matter and organic compounds that could contaminate the final product. The effect of the bittern composition and salinity was also investigated. These bittern features and their COD are significantly dependent on the sampling season. For the investigated pilot, the effect of pressure, composition and organic content on permeability is significant.

The fouling effect of bittern was also investigated by finding resistance coefficients ($R_{f}$) 2 orders of magnitude higher than those found for the same application but with river water in different UF plants reported in the literature.

A reduction in the salt concentration of permeate and retentate compared to the feed was found, confirming previous works in the literature on brines and also extending their validity to bitterns. Moreover, for the case of the CS-2 bittern, a rejection rate of 95% was achieved working at 2 bar. This was identified as the best operating condition for this plant, as it also guarantees a higher permeate permeability value and lower fouling growth. In addition, under this operating condition, the ionic composition of the permeate did not exhibit large variations.

Moreover, an empirical rheological model was developed in order to calculate the viscosity of the bittern at different temperatures and magnesium concentrations. The model obtained a ±5% error in predicting viscosity for bitterns with Mg concentration up to 2.5 M. Also, the viscosity–temperature trend was properly predicted with an average error of 5%.

A long run test was also carried out, showing good reliability of the plant over time.

The investigation performed on this pilot-scale UF plant provides very important information for the characterization of this technology in the treatment and valorization of hypersaline solutions, enabling the recovery of minerals in a sustainable and efficient manner. Furthermore, the ability to accurately predict the viscosity of bitterns based on temperature and magnesium concentration provides a model applicable on a large scale for designing industrial facilities for the treatment of real bitterns for mineral recovery. The experimental findings collected in this work, filling such gaps in the current literature, could accelerate the development of innovative solutions for the industrial scale-up of the proposed concept, fostering new opportunities for resource recovery from bitterns and reducing the associated cost.

## Figures and Tables

**Table 1:** The average ionic composition of seawater and bitterns collected from different sites within the Mediterranean basin.

Ion	Seawater (mg/L) [3,4]	Bittern (mg/L) [5]
Chloride (Cl^−^)	19,000–22,000	175,000–190,000
Sodium (Na^+^)	10,500–12,500	44,000–84,000
Sulfate (SO_4_^2−^)	2650–3450	35,000–60,000
Magnesium (Mg^2+^)	1250–1400	20,000–60,000
Potassium (K^+^)	380–460	6500–14,000
Bromide (Br^−^)	8–155	880–2500
Calcium (Ca^2+^)	400–425	60–150
Boron (B^3+^)	1–5	85–200
Strontium (Sr^2+^)	0–10	10–25
Lithium (Li^+^)	0.1–0.2	3–10

**Table 2:** Composition of three samples of Trapani saltworks’ bitterns analyzed in the present study.

	Case Study 1 (CS-1) (Summer)	Case Study 2 (CS-2) (Autumn)	Case Study 3 (CS-3) (Spring)
Na^+^ [g/L]	52.5 ± 0.49	33.58 ± 0.27	65.36 ± 0.18
K^+^ [g/L]	13.7 ± 0.03	15.02 ± 0.34	6.77 ± 0.05
Ca^2+^ [g/L]	0.15 ± 0.01	0.082 ± 0.01	0.326 ± 0.01
Mg^2+^ [g/L]	50.28 ± 0.10	59.90 ± 1.11	23.70 ± 0.10
B^3+^ [mg/L]	191.27 ± 3.23	203.15 ± 5.15	95.22 ± 1.12
Cl^−^ [g/L]	163.5 ± 2.5	164.03 ± 2.02	163.19 ± 0.98
SO_4_^2−^ [g/L]	57.86 ± 0.12	72.18 ± 0.68	33.34 ± 0.65
Br^−^ [g/L]	1.67 ± 0.08	2.21 ± 0.07	1.27 ± 0.05
Conductivity [mS/cm]	144.2	147.1	193.4
pH [-]	7.1	6.6	7.6
Density at 25 °C [g/L]	1259	1291	1249
Viscosity at 25 °C [cP]	5.06	6.69	3.38
COD [mg/L]	1285	1033	831

**Table 3:** Coefficients of the viscosity model after calibration with experimental data.

$A_{1}\;\left[\frac{L^{1/2}}{{\mathrm{mol}}^{1/2}}\right]$	$A_{2}\;\left[\frac{L}{\mathrm{mol}}\right]$	$A_{3}\;\left[\frac{L^{2}}{{\mathrm{mol}}^{2}}\right]$	$A_{4}\;\left[\frac{L}{\mathrm{mol}}\right]$
−0.3744	2.7691	−0.2291	−0.1847

## Data Availability

The raw data supporting the conclusions of this article will be made available by the authors on request.

## Nomenclature

$L_{p}$	$Membrane\;Permeability\;\left[{L\;m}^{-2}h^{-1}{\mathrm{bar}}^{-1}\right]$
$C_{org_{feed}}$	$Organic\;compound\;concentration\;in\;feed\;stream\;\left[{\mathrm{mg}\;L}^{-1}\right]$
$C_{org_{per}}$	$Organic\;compound\;concentration\;in\;permeate\;stream\;\left[{\mathrm{mg}\;L}^{-1}\right]$
$J_{v}$	$Volumetric\;flux\;\left[{L\;m}^{-2}h^{-1}\right]$
$P_{feed}$	$Feed\;Pressure\;\left[\mathrm{bar}\right]$
$P_{perm}$	$Permeate\;Pressure\;\left[\mathrm{bar}\right]$
$P_{ret_{out}}$	$Retentate\;Pressure\;\left[\mathrm{bar}\right]$
$R_{f}$	$Fouling\;Resistance\;\left[m^{-1}\right]$
$R_{m}$	$Membrane\;Resistance\;\left[m^{-1}\right]$
ΔP	$Pressure\;difference\;\left[\mathrm{bar}\right]$
R	Organic Rejection
μ	$Dynamic\;Viscosity\;\left[\mathrm{cP}\right]$
ν	$Kinematic\;Viscosity\;\left[\mathrm{Cst}\right]$
ρ	$Density\;\left[{\mathrm{kg}\;m}^{-3}\right]$

## Abbreviations

COD	Chemical Oxygen Demand
CS	Case Study
DSB	Dead Sea Brine
EDBM	Electro-Dialysis with Bipolar Membrane
IEP	Iso-Electric Point
MF	Microfiltration
MG-CGCR	Magnesium–Cristal Granulometry Controlled Reactor
MED	Multiple Effect Distillation
MSE	Mean Squared Error
MSF	Multiple Stages Flash
MVC	Mechanical Vapor Compressor
NF	Nanofiltration
NTU	Nephelometric Turbidity Unit
pHSA	pH Swing Adsorption
TMP	Trans-Membrane Pressure
TRL	Technology Readiness Level
UF	Ultrafiltration
